# Roles of N-linked and O-linked glycosylation sites in the activity of equine chorionic gonadotropin in cells expressing rat luteinizing hormone/chorionic gonadotropin receptor and follicle-stimulating hormone receptor

**DOI:** 10.1186/s12896-021-00712-8

**Published:** 2021-09-05

**Authors:** So-Yun Lee, Munkhzaya Byambaragchaa, Seung-Hee Choi, Han-Ju Kang, Myung-Hwa Kang, Kwan-Sik Min

**Affiliations:** 1 Animal Biotechnology, Graduate School of Future Convergence Technology, Institute of Genetic Engineering, Hankyong National University, Ansung, 17579 Korea; 2 Department of Food Science and Nutrition, Hoseo University, Asan, 31499 Korea; 3 School of Animal Life Biotechnology, Institute of Genetic Engineering, Hankyong National University, Ansung, 17579 Korea

**Keywords:** Recombinant-equine chorionic gonadotropin, Glycosylation sites, cAMP responses, CHO-suspension cells, Rat LH/CG receptor, Rat FSH receptor

## Abstract

**Background:**

Equine chorionic gonadotropin (eCG), which comprises highly glycosylated α-subunit and β-subunit, is a unique member of the glycoprotein hormone family as it elicits both follicle-stimulating hormone (FSH)-like and luteinizing hormone (LH)-like responses in non-equid species. To examine the biological function of glycosylated sites in eCG, the following glycosylation site mutants were constructed: eCGβ/αΔ56, substitution of Asn^56^ of α-subunit with Gln; eCGβ-D/α, deletion of the O-linked glycosylation site at the carboxyl-terminal peptide (CTP) region of the β-subunit; eCGβ-D/αΔ56, double mutant. The recombinant eCG (rec-eCG) mutants were expressed in Chinese hamster ovary suspension (CHO-S) cells. The FSH-like and LH-like activities of the mutants were examined using CHO-K1 cells expressing rat lutropin/CG receptor (rLH/CGR) and rat FSH receptor (rFSHR).

**Results:**

Both rec-eCGβ/α and rec-eCGβ/αΔ56 were efficiently secreted into the CHO-S cell culture medium on day 1 post-transfection. However, the secretion of eCGβ-D/α and eCGβ-D/αΔ56, which lack approximately 12 O-linked glycosylation sites, was slightly delayed. The expression levels of all mutants were similar (200–250 mIU/mL) from days 3 to 7 post-transfection. The molecular weight of rec-eCGβ/α, rec-eCGβ/αΔ56 and rec-eCG β-D/α were in the ranges of 40–45, 37–42, and 34–36 kDa, respectively. Treatment with peptide-N-glycanase F markedly decreased the molecular weight to approximately 5–10 kDa. Rec-eCGβ/αΔ56 exhibited markedly downregulated LH-like activity. The signal transduction activity of both double mutants was completely impaired. This indicated that the glycosylation site at Asn^56^ of the α-subunit plays a pivotal role in the LH-like activity of eCG. Similarly, the FSH-like activity of the mutants was markedly downregulated. eCGβ-D/α exhibited markedly downregulated LH-like and FSH-like activities.

**Conclusions:**

Rec-eCGβ/α exhibits potent biological activity in cells expressing rLH/CGR and rFSHR. The findings of this study suggest that the LH-like and FSH-like activities of eCG are regulated by the N-linked glycosylation site at Asn^56^ of the eCG α-subunit and/or by the O-linked glycosylation sites of the eCG β-subunit. These findings improved our understanding of the mechanisms underlying both LH-like and FSH-like activities of eCG.

## Background

Equine chorionic gonadotropin (eCG), a unique member of the gonadotropin family, exhibits both luteinizing hormone (LH)-like and follicle-stimulating hormone (FSH)-like activities in non-equid species [1–5]. CG was reported to be expressed only in primate and Equidae species during early pregnancy. In particular, eCG is secreted from the endometrial cups that detach from the chorionic girdle of the conceptus between days 37 and 120 of pregnancy [6–8]. The administration of eCG increases the ovulation rate [9, 10], especially in early post-partum cows [11] and cows under prolonged anestrus or seasonal heat stress [9]. The combination of eCG and human CG (hCG) is used to induce ovulation in experimental animals [12, 13]. In sheep, the oocytes matured in the presence of eCG (20 µg/mL) exhibited significantly higher FSH receptor (FSHR) levels than those matured in the absence of eCG [14]. However, eCG administration between days 9 and 15 post-partum did not significantly affect the reproductive performance as evidenced by the lack of correlation between eCG treatment and parity and milk yield [15].

A study of recombinant eCG (rec-eCG) revealed that the amino acid (aa) residues 102–104 of the β-subunit are critical for the binding of eCG to FSHR [16]. Additionally, the aa residues 104–109 of the β-subunit are critical for the secretion of a fully folded eCGβ/α and its FSH-like activity but are not critical for its LH-like activity [4]. Previously, we had reported that rec-eCG produced from CHO-K1 cells exhibited both LH-like and FSH-like activities in the rat Leydig cells, rat granulosa cells [17], and cells expressing rat lutropin/CG receptor (rLH/CGR) and rat FSHR [18–20]. Recently, we demonstrated that the administration of rec-eCG proteins significantly decreased the number of nonfunctional oocytes and that the frequency of nonfunctional oocytes in the natural eCG-treated and rec-eCG-treated groups was approximately 20% and 2%, respectively [13]. Additionally, the microarray analysis revealed the differential expression of ovulation-related genes in mouse ovary between the rec-eCG-treated and natural eCG-treated groups [21].

The eCG α-subunit contains two N-linked glycosylation sites at aa residues Asn^56^ and Asn^82^. Meanwhile, the β-subunit of eCG contains one N-linked glycosylation site at Asn^13^ and approximately 11 O-linked glycosylation sites in the carboxyl-terminal peptide (CTP) region [22–24]. eCG has the highest carbohydrate content (more than 40%) among all known glycoprotein hormones. A single gene encodes the β-subunits of eCG and eLH [25]. Equine CG and eLH possess the same dual biological activities, but differ in carbohydrate composition [26, 27]. Previous studies on hCGαΔ52/β have revealed that the N-linked oligosaccharide site at Asn^52^ of the α-subunit is essential for the LH-like activity of hCG [28, 29]. Similarly, studies on hFSHαΔ52/β (mutated N-linked glycosylation site at Asn^52^ of α-subunit) have revealed that Asn^52^ of the α-subunit is critical for the biological activity of hFSH [30–32]. eFSHαΔ56/β with mutation at the Asn^56^ of the α-subunit does not exhibit FSH-like activity. This indicated that the oligosaccharide at Asn^56^ was necessary for the function of eFSH in rat granulosa cells [33]. Thus, the N-linked glycosylation sites at Asn^52^ of the hCG/hFSH α-subunit and Asn^56^ of the eFSH α-subunit have an indispensable role in LH-like and FSH-like signal transduction.

Previously, we examined the different roles of rec-eCG and its glycosylation patterns in primary cultures of rat Leydig cells and granulosa cells [17, 33, 34]. The LH-like activity of αΔ56/β was completely downregulated during testosterone production in rat Leydig cell culture. However, the FSH-like activity of eCGαΔ56/β was similar to that of wild-type eCG, which indicated that the eCGαΔ56/β mutant stimulated estradiol production in the primary culture of rat granulosa cells. Thus, the biological roles of glycosylation site at Asn^56^ of the eCG α-subunit appear to be different in both LH-like and FSH-like activities in primary cultures of rat Leydig cells and granulosa cells. However, the effects of glycosylation site mutation on LH-like and FSH-like activities of eCG-mediation stimulation of cAMP response have not been examined in cells expressing rLH/CGR and rFSHR.

This study aimed to delineate the roles of glycosylation sites in LH-like and FSH-like activities of eCG using rec-eCG mutants. Single-chain forms of eCG and its glycosylation site mutants were constructed and the biological activity of these proteins was examined in vitro using cells expressing rLH/CGR and rFSHR. The findings of this study revealed the role of glycosylation sites in LH-like and FSH-like activities of eCG.

## Results

### Secretion of rec-eCG mutants in the CHO-S cell culture mediums

**Fig. 1:**
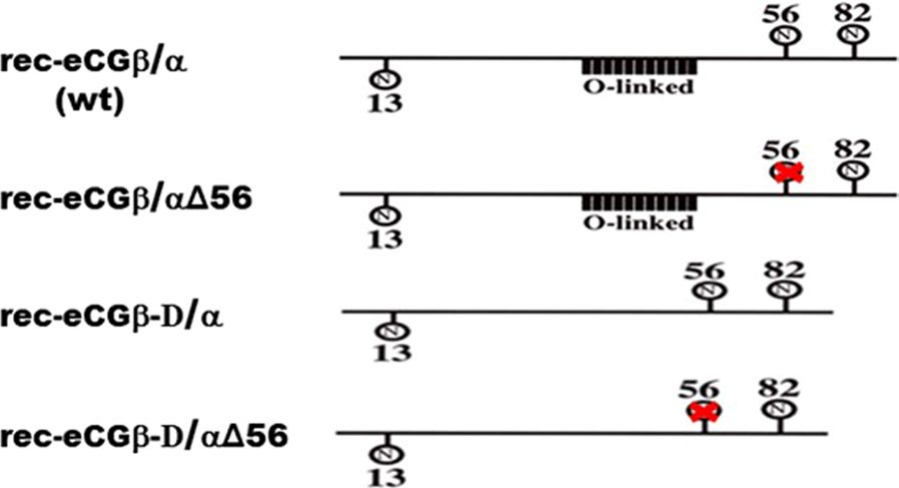
Schematic diagram of wild-type and mutant recombinant equine chorionic gonadotropin (rec-eCG). The wild-type and mutant N-linked and O-linked glycosylation sites on eCG. Asn^56^ of the eCG α-subunit was replaced with Gln or the carboxyl-terminal peptide (CTP) region of O-linked oligosaccharides in the eCG β-subunit was deleted using polymerase chain reaction. The circles “N,” “X,” and “O-linked” denote N-linked oligosaccharide, non-glycosylated sites, and O-linked oligosaccharide at the eCG β-subunit, respectively. The four expression vectors were constructed (plasmids encoding eCGβ/α-wt and mutants designated as pcDNA3-eCGβ/α, pcDNA-eCGβ/αΔ56, pcDNA-eCGβ-D/α, and pcDNA3-eCGβ-D/αΔ56. eCGβ-D/αΔ56 implies a double mutant with substitution of Asn^56^ of eCG α-subunit and deletion at the CTP region of the eCG β-subunit. The epitope *myc*-tags was inserted between the first and second amino acid residues of the β-subunit of the mature eCG protein

**Fig. 2:**
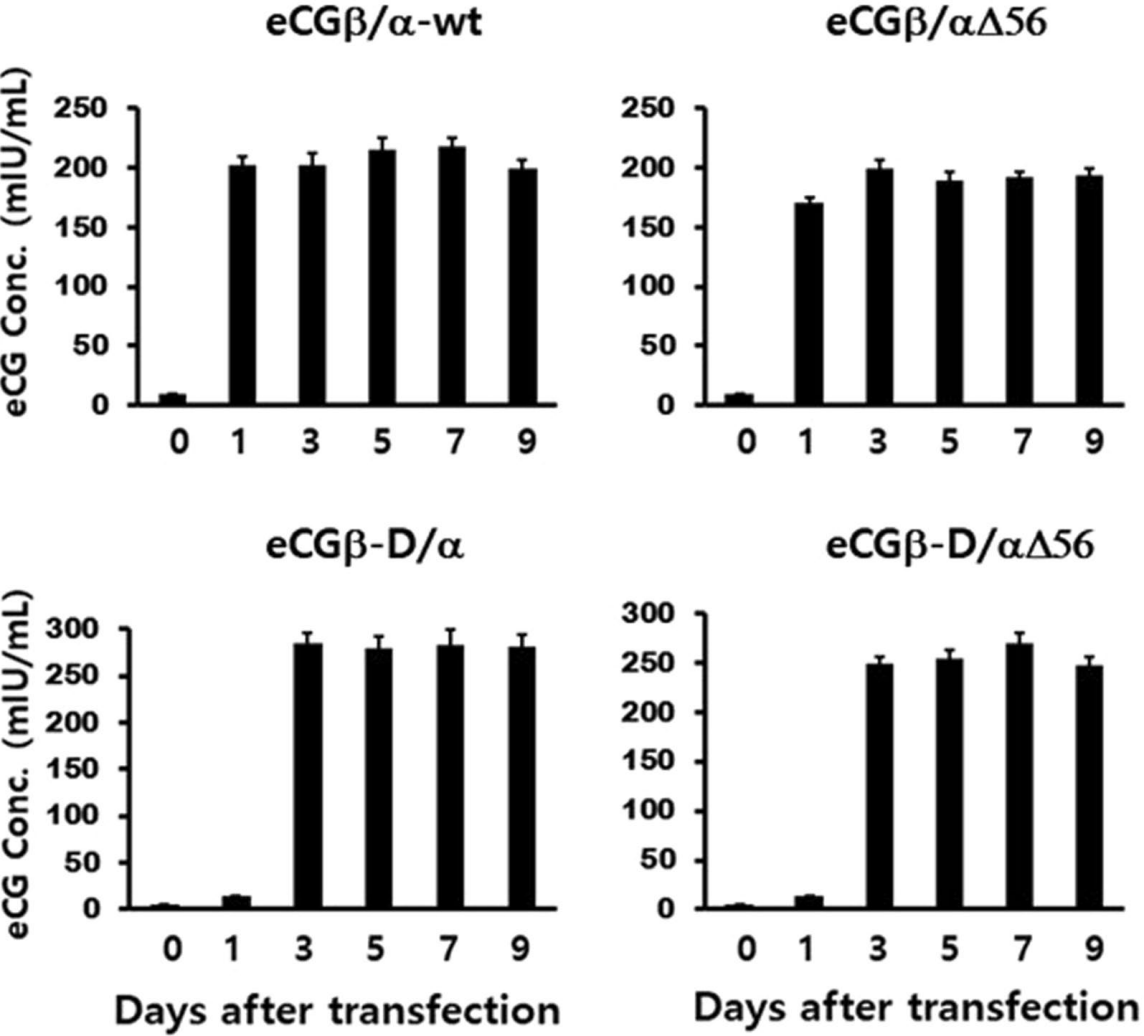
Quantification of recombinant equine chorionic gonadotropin (rec-eCG) proteins in Chinese hamster ovary cell suspension. The culture media were collected and centrifuged on days 0, 1, 3, 5, 7, and 9 post-transfection. The expression level of rec-eCGβ/α proteins was analyzed using enzyme-linked immunosorbent assay as described in the Methods section. Values are expressed as mean ± standard error of mean from at least three independent experiments

Site-directed mutagenesis was performed to examine the importance of glycosylation sites in LH-like and FSH-like activities of rec-eCG. The following four recombinant expression vectors were constructed in this study: rec-eCGβ/α (rec-eCG-wt), rec-eCGβ/αΔ56, rec-eCGβ-D/α, and rec-eCGβ-D/αΔ56 (Fig. 1). The levels of rec-eCG proteins in the culture supernatant of CHO-S cells transfected with rec-eCG-wt, rec-eCGβ/αΔ56, rec-eCGβ-D/α, or rec-eCGβ-D/αΔ56 constructs were measured on days 0–9 post-transfection. As shown in Fig. 2, the level of rec-eCG β/α-wt was more than 200 mIU/mL on day 1 post-transfection, which was maintained till day 9 post-transfection. The expression level of rec-eCGβ/αΔ56 was similar to that of rec-eCG β/α-wt although the expression level of rec-eCGβ/αΔ56 on day 1 post-transfection (170 ± 5 mIU/mL) slightly lower than that of rec-eCGβ/α-wt. rec-eCGβ-D/α and rec-eCGβ-D/αΔ56 were not detected on day 1 post-transfection. However, the expression levels of rec-eCGβ-D/α and rec-eCGβ-D/αΔ56 markedly increased to 285 ± 11 and 250 ± 7 mIU/mL on day 3 post-transfection, respectively. The expression levels of rec-eCGβ-D/α and rec-eCGβ-D/αΔ56 in spinner flask cultures were more than 250 mIU/mL till day 9 post-transfection. Thus, the secretion of CTP deletion mutants (eCGβ-D/α and eCGβ-D/αΔ56) was delayed when compared with that of eCGβ/α-wt and eCGβ/αΔ56. The levels of rec-eCGβ-D/α and eCGβ-D/αΔ56 were slightly higher than those of rec-eCGβ/α-wt from day 3 post-transfection. These findings indicated that the CTP region containing approximately 12 O-linked glycosylation sites in eCG β-subunit and eLH β-subunit is critical for the early secretion into the culture medium of CHO-S cells.

### Western blot analysis

**Fig. 3:**
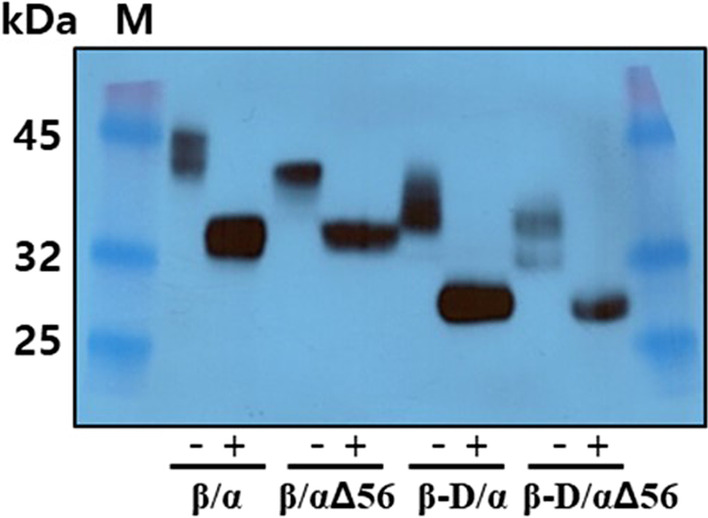
Western blotting analysis of recombinant equine chorionic gonadotropin (rec-eCG). The proteins in the conditioned media were collected and concentrated 5–10-fold. The rec-eCG samples were resolved using sodium dodecyl sulfate–polyacrylamide gel electrophoresis and blotted onto a membrane. The proteins were detected using anti-*myc*-tag and horseradish peroxidase-conjugated goat anti-mouse IgG antibodies. The proteins were treated with peptide-N-glycanase F to remove N-linked oligosaccharides and subjected to western blotting. Lane 1, Marker; Lane 2, rec-eCGβ/α-wt; Lane 3, rec-eCGβ/αΔ56, Lane 4, rec-eCGβ-D/α; Lane 5, rec-eCGβ-D/αΔ56, − , not treated with peptide-N-glycanase F; +, treated with peptide-N-glycanase F

Next, the molecular weight of rec-eCG proteins was examined using western blotting analysis. The apparent molecular weight of natural eCG ranges from 50 to > 100 kDa, rec-eCG is much smaller due to hypoglycosylation by CHO cells. The molecular weight of rec-eCGβ/α-wt was in the range of 40–45 kDa. Meanwhile, the molecular weight of the rec-eCGβ/αΔ56, which does not contain one N-linked glycosylation site at Asn^56^ of the α-subunit, was in the range of 37–42 kDa (Fig. 3). This indicated that the molecular weight of one N-linked glycosylation site was approximately 3–4 kDa. Treatment with peptide-N-glycanase F decreased the molecular weight of both rec-eCGβ/α-wt and rec-eCGβ/αΔ56 to approximately 32–34 kDa. The molecular weight of rec-eCGβ-D/α, which lacks the 35 aa residues comprising the O-linked glycosylation sites of the eCG β-subunit, was approximately 35–37 kDa. Treatment with peptide N-glycanase F decreased the molecular weight of both rec-eCGβ-D/α and rec-eCGβ-D/αΔ56 to approximately 26–28 kDa. The molecular weight of rec-eCGβ/α-wt markedly decreased to approximately 10–12 kDa upon treatment with peptide N-glycanse F. These results indicate that rec-eCGβ/α and its mutants exhibit differential glycosylation patterns in CHO-S cells and that the N-linked glycosylation site mutants were not glycosylated rather than wild type eCGβ/α. Thus, CHO-S cells are a good host for the production of recombinant glycoproteins with partially glycosylated, but not like glycosylation structure of natural glycoproteins.

### LH-like activity of rec-eCG mutants in cells expressing rLH/CGR

**Fig. 4:**
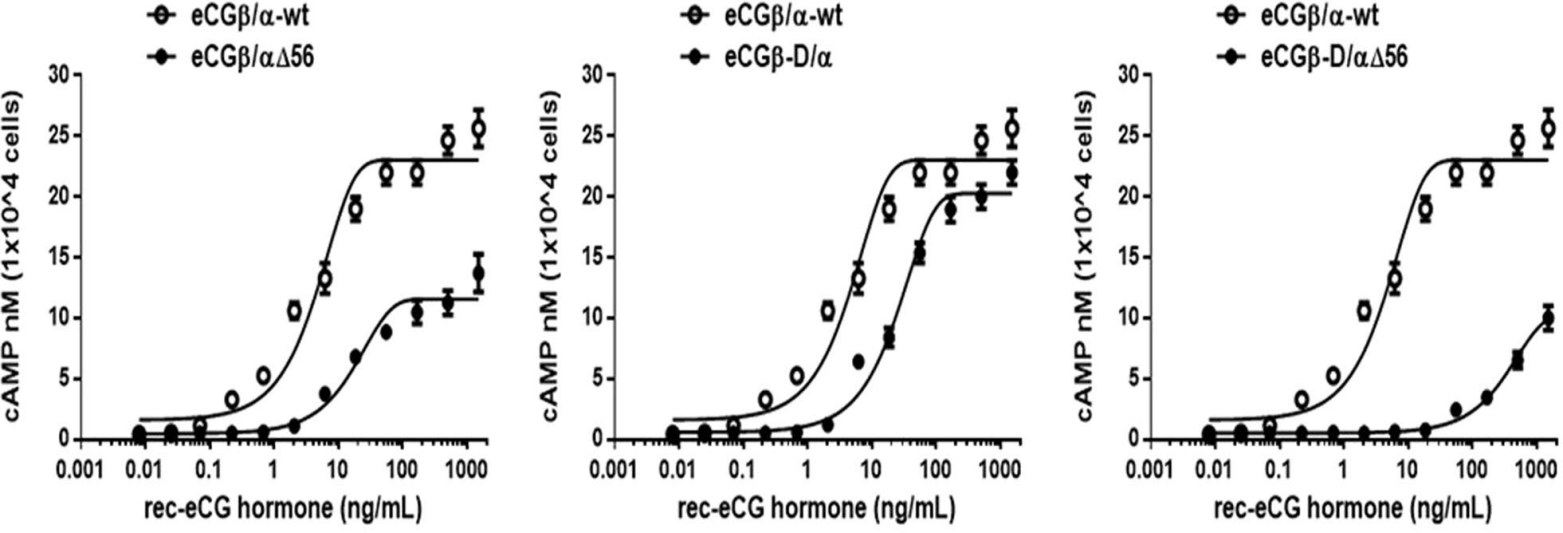
Effect of wild-type and mutant recombinant equine chorionic gonadotropin (rec-eCG) on cyclic adenine monophosphate (cAMP) production in cells expressing rat lutropin/chorionic gonadotropin receptor (rLH/CGR). Cells transiently transfected with rLH/CGR were seeded in 384-well plates (10,000 cells per well) at 24 h post-transfection. The cells were incubated with re-eCG proteins for 30 min at room temperature. cAMP production was detected using a homogeneous time-resolved fluorescence assay and was represented as Delta F%. The cAMP concentrations were calculated using GraphPad Prism software. The results of the mock-transfected cells were subtracted from each dataset (see Methods). Each data point represents mean ± standard error of mean from triplicate experiments. The mean data were fitted to the equation of a one-phase exponential decay curve. The blank circles show the data of the wild-type receptor

The in vitro LH-like activity of rec-eCG mutants was assessed using CHO-K1 cells expressing rLH/CGRR. The ability of rec-eCG mutants to activate the production of cAMP is shown in Fig. 4. Rec-eCGβ/α-wt dose-dependently increased the concentration of cAMP. This is indicated that rec-eCGβ/α-wt produced in the CHO-S cells expressing rLH/CGR can elicit a LH-like biological response in vitro. The half-maximal effective concentration (EC_50_) and Rmax values of rec-eCGβ/α-wt for the production of cAMP were 4.6 ng/mL and 23.1 ± 0.5 nM/10^4^ cells, respectively (Table 1). The dose–response curve of rec-eCGβ/αΔ56 markedly shifted to the right. The EC_50_ value of the rec-eCGβ/αΔ56 for the production cAMP was approximately 3.8-fold (17.63 ng/mL) lower than that of rec-eCGβ/α-wt. The Rmax value of rec-eCGβ/αΔ56 was approximately 11.6 ± 0.3 nM/10^4^ cells. Comparative analysis of rec-eCGβ/α-wt and the mutants confirmed the specificity of the glycosylation site at Asn^56^ of the α-subunit. The LH-like activity of rec-eCGβ-D/α was 5.3-fold lower than that of rec-eCGβ/α-wt. The Rmax value of rec-eCGβ-D/α was approximately 0.88-fold (20.3 ± 0.4 nM/10^4^ cells) lower than that of rec-eCGβ/α. The dose–response curve of the double mutant (rec-eCGβ-D/αΔ56) markedly shifted to the right. The EC_50_ value of rec-eCGβ-D/αΔ56 (334.2 ng/mL) was 72.7-fold lower than that of rec-eCGβ/α-wt. The Rmax value of the double mutant (10.3 ± 0.4 nM/10^4^ cells) was 0.45-fold lower than that of rec-eCGβ/α.

**Table 1:** Bioactivity of rec-eCG mutants in cells expressing ratLH/CG receptor

rec-eCG	cAMP responses		
	Basal^a^ (nM/10^4^ cells)	EC_50_^b^ (ng/mL)	Rmax^c^ (nM/10^4^ cells)
rec-eCGβ/α-wt	0.4 ± 0.1	4.6 (1.0-fold) (3.6 to 6.3)^d^	23.1 ± 0.5 (1.0-fold)
rec-eCGβ/αΔ56	0.3 ± 0.1	17.63 (3.8-fold) (13.9 to 24.1)	11.6 ± 0.3 (0.50-fold)
rec-eCGβ-D/α	0.3 ± 0.1	24.5 (5.3-fold) (20.9 to 29.6)	20.3 ± 0.4 (0.88-fold)
rec-eCGβ-D/αΔ56	0.2 ± 0.1	334.2 (72.7-fold) (282.4 to 409.1)	10.3 ± 0.4 (0.45-fold)

Values are the means ± SEM of triplicate experiments. EC_50_ values were determined from the concentration–response curves from in vitro bioassays. The cAMP responses of the basal and Rmax in rec-eCGβ/α-wild type were shown as onefold

^a^Basal cAMP level average without agonist treatment

^b^Geometric mean (95% confidence limit)

^c^Rmax average cAMP level/10^4^ cells

^d^95% Confidence intervals

Thus, the glycosylation sites at Asn^56^ of the α-subunit and in the CTP of the β-subunit co-operatively promote LH-like activity. The dose–response curve of rec-eCGβ/αΔ56 markedly shifted to the right. Additionally, rec-eCGβ/αΔ56 exhibited decreased activity, which indicated that the glycosylation site at Asn^56^ of the α-subunit is indispensable for the LH-like activity of eCG. The LH-like activity of rec-eCGβ-D/αΔ56 was markedly downregulated, which demonstrated that the glycosylation sites at Asn^56^ of α-subunit and CTP of β-subunit were essential for the LH-like activity of eCG.

### FSH-like activity of rec-eCG mutants in cells expressing rFSHR

**Fig. 5:**
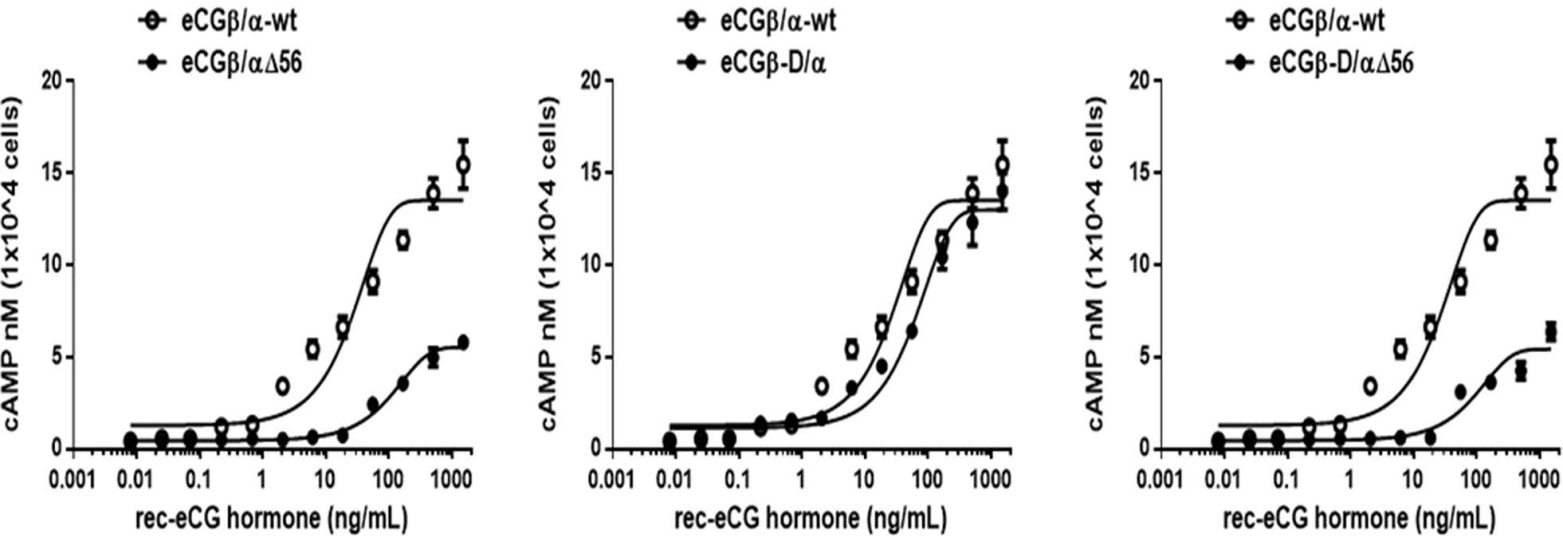
Effect of wild-type and mutant recombinant equine chorionic gonadotropin (rec-eCG) on total cyclic adenine monophosphate (cAMP) levels in the Chinese hamster ovary (CHO-K1) cells transfected with rat follicle-stimulating hormone receptor (rFSHR). rec-eCGβ/α proteins dose-dependently increased cAMP accumulation in CHO-K1 cells transiently transfected with rFSHR. CHO-K1 cells were transfected with rFSHR and the production of cAMP in the cells was analyzed at 48–72 h post-transfection (see Methods for details). The cAMP values were calculated using GraphPad Prism

**Fig. 6:**
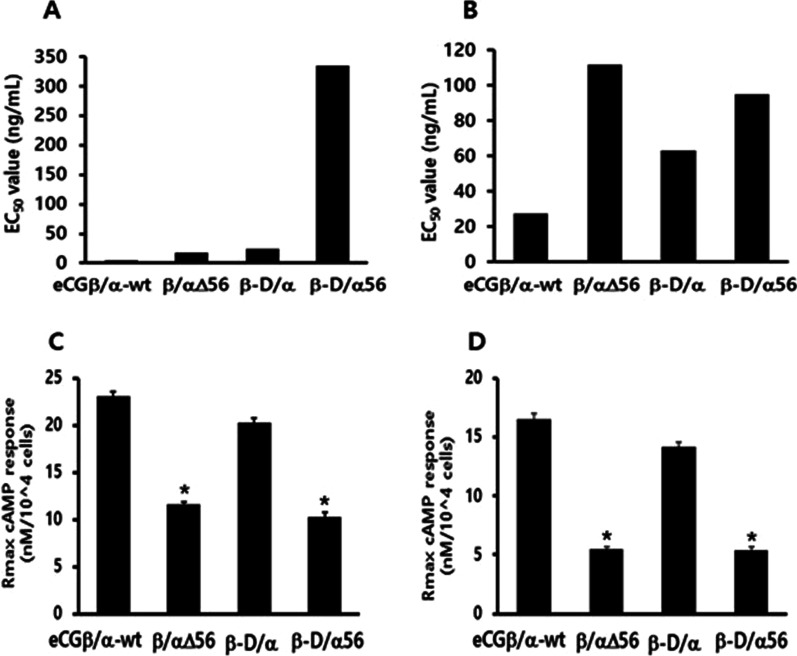
Effect of wild-type and mutant recombinant equine chorionic gonadotropin (re-eCG) on half maximal effective concentration (EC_50_) value and maximal response (Rmax) level in Chinese hamster ovary (CHO-K1) cells transfected with rat luteinizing hormone/chorionic gonadotropin receptor (rLH/CGR) and rat follicle-stimulating hormone receptor (rFSHR). The EC_50_ value in cells expressing rLH/CGR (**A**) and cells expressing rFSHR (**B**). The Rmax values of wild-type and mutant rec-eCGβ/α in cells expressing rLH/CGR (**C**) and cells expressing rFSHR (**D**). Data are expressed as mean ± standard error of mean from triplicate experiments. Values with asterisks are significantly different (*P* < 0.05)

The in vitro FSH-like activity of rec-eCG mutants was assessed using CHO-K1 cells expressing rFSHR. The cells were incubated with rec-eCG mutants and the production of cAMP was measured. rec-eCGβ/α-wt dose-dependently increased the concentration of cAMP (Fig. 5). The EC_50_ and Rmax values of rec-eCGβ/α-wt for FSH-like activity were 27.1 ng/mL and 16.5 ± 0.5 nM/10^4^ cells, respectively. The dose–response curves of all mutants markedly shifted to the right. In particular, the EC_50_ values of eCGβ/αΔ56 and eCGβ-D/αΔ56 were 111.5 and 94.9 ng/mL, respectively (Table 2). This indicated that the activities of eCGβ/αΔ56 and eCGβ-D/αΔ56 were 4.1-fold and 3.5-fold lower than those of rec-eCGβ/α-wt, respectively. The Rmax values of eCGβ/αΔ56 and eCGβ-D/αΔ56 were 5.5 ± 0.2 and 5.4 ± 0.3 nM/10^4^ cells. The EC_50_ and Rmax values of rec-eCGβ-D/α were 2.3-fold (63.1 ng/mL) higher and 0.86-fold (14.2 ± 0.4 nM/10^4^ cells) lower than those of rec-CGβ/α-wt, respectively. The FSH-like activity of rec-eCG is dependent on glycosylation site at Asn^56^ of the eCG α-subunit. The EC_50_ and Rmax values of eCGβ/αΔ56 and eCGβ-D/αΔ56 were markedly lower than those of eCGβ/α-wt. In particular, the EC_50_ values of eCGβ/αΔ56 and eCGβ-D/αΔ56 were 24.3% and 28.5% of those of rec-eCGβ/α-wt, respectively (Fig. 6). Similarly, the Rmax values of eCGβ/αΔ56 and eCGβ-D/αΔ56 were 33.3% and 32.7% of those of eCGβ/α-wt (Fig. 6). The glycosylation site at Asn^56^ of eCG α-subunit plays a pivotal role in signal transduction. Hence, mutation at this site markedly decreased both LH-like and FSH-like activities. The Rmax values of of eCGβ/αΔ56 and eCGβ-D/αΔ56 were markedly lower than those of rec-eCGβ/α-wt and rec-eCGβ-D/α (Fig. 6).

**Table 2:** Bioactivity of rec-eCG mutants in cells expressing ratFSH receptor

rec-eCG	cAMP responses		
	Basal^a^ (nM/10^4^ cells)	EC_50_^b^ (ng/mL)	Rmax^c^ (nM/10^4^ cells)
rec-eCGβ/α-wt	0.4 ± 0.1	27.1 (1.0-fold) (20.2 to 40.8) ^d^	16.5 ± 0.5 (1.0-fold)
rec-eCGβ/αΔ56	0.4 ± 0.1	111.5 (4.1-fold) (94.8 to 135.2)	5.5 ± 0.2 (0.33-fold)
rec-eCGβ-D/α	0.3 ± 0.1	63.1 (2.3-fold) (51.3 to 81.9)	14.2 ± 0.4 (0.86-fold)
rec-eCGβ-D/αΔ56	0.4 ± 0.1	94.9 (3.5-fold) (69.8 to 147.8)	5.4 ± 0.3 (0.32-fold)

Values are the means ± SEM of triplicate experiments. EC_50_ values were determined from the concentration–response curves from in vitro bioassays. The cAMP responses of the basal and Rmax in rec-eCGβ/α-wild type were shown as onefold

^a^Basal cAMP level average without agonist treatment

^b^Geometric mean (95% confidence limit)

^c^Rmax average cAMP level/10^4^ cells

^d^95% Confidence intervals

These findings suggest that the CTP region of the eCG β-subunit is not as important as the glycosylation site at Asn^56^ of eCG α-subunit for FSH-like activity. However, the glycosylation sites in the CTP region of the eCG β-subunit were essential for the FSH-like activity of eCG. The EC_50_ values of eCGβ-D/α and eCGβ-D/αΔ56 were 42.9% and 28.5% of those of eCGβ/α-wt. Thus, these glycosylation sites are critical for signal transduction through rFSHR. This study, for the first time, demonstrated the signal transduction of rec-eCG mutants in cells expressing rLH/CGR and rFSHR. Additionally, this study demonstrated that rec-eCG mutants exhibit potent biological activity in cells expressing rLH/CGR and rFSHR, which can aid in devising strategies to regulate the biological activity of glycosylation site rec-eCG mutants.

## Discussion

N-linked glycosylation sites mediate several functions, including protein secretion, receptor binding, and biological activity [35, 36]. The biological function of the glycosylation sites of glycoprotein hormones (gonadotropins) has been examined using site-directed mutagenesis [28–30, 37–39]. Single-chain gonadotropins in which the α-subunit is fused to the CTP of the β-subunit exhibit potent biological activities [35, 40, 41]. Recently, we reported that the glycosylation sites play an essential role in the biological activity of eel FSH [42], eel LH [43], and eCG [13, 21]. The functions of each glycosylation site in rec-eCG have been examined in the primary rat Leydig cells and rat granulosa cells [17, 34] but not in the cells expressing rLH/CGR and rFSHR. Additionally, the role of glycosylation sites in the ovulation rate in mouse ovaries was examined through comparative gene expression profiling of glycosylation mutants [13, 21].

In this study, glycosylation site mutants (substitution of Asn^56^ with Gln in the eCG α-subunit and deletion of the O-linked glycosylated sites of the eCG β-subunit) were constructed using site-

directed mutagenesis to investigate the biological functions of glycosylation sites in single-chain eCG by site-directed mutagenesis. rec-eCGβ/α-wt was efficiently secreted into the culture medium of the CHO-S cells on day 1 post-transfection. Additionally, the results of this study indicated that the CTP region (114–149 aa) of the eCG β-subunit containing approximately 12 O-linked glycosylation sites was critical for the early secretion of eCG into the culture medium of CHO-S cells. This is consistent with the secretion rate of hCG lacking CTP (114–145 aa), which was three-fold to four-fold prolonger than that of hCG-wt containing the CTP [40]. A pulse-chase analysis revealed that the secretion of an hFSH single-chain mutant (hFcα) in which the CTP region of the hCG β-subunit (118–145 aa) was inserted between the hFSH β-subunit and the α-subunit was markedly rapid when compared with that of hFSH-wt [41]. Transfection with only FSH β-subunit resulted in slow secretion, which was similar to the secretion kinetics of LH β-subunit. However, the hCG β-subunit is efficiently and rapidly secreted [44]. Consistent with these observations, rec-eCG β-D/α and rec-eCGβ-D/αΔ56 were detected in the culture supernatant on day 3 post-transfection in this study. Thus, the CTP linker (114–149 aa) of the eCG β-subunit plays an important role in the efficient secretion of eCG.

In the present study, rec-eCGβ/αΔ56 mutation did not affect the secretion or the expression of the protein. This is consistent with the loss of glycosylation site at the aa residue 52 of the hCG α-subunit, which indicated that the kinetics of secretion and recovery of mutant protein were identical to those of wild-type hCG [37]. Only 17% of the synthesized Asn^78^ mutants were recovered, which suggested that the loss of carbohydrate at Asn^78^ affects the stability and promotes degradation [38]. Based on our previous studies on eel LH and FSH, we hypothesized that the Asn^56^ residue of the α-subunit did not affect the secretion into the medium. However, the secretion of Asn^79^ mutant of the α-subunit was significantly lower than that of the wild-type [42, 43]. Previously, we had also reported that the specific glycosylated sites (Asn^82^ of the α-subunit and Asn^13^ of the β-subunit) are critical for the secretion of the rec-eCGα/β dimer and single-chain rec-eCGβ/α [13]. Therefore, we assumed that the CTP region of the eCG β-subunit was more important than Asn^56^ of the eCG α-subunit for protein secretion. The mechanism involved in the delayed secretion of eCG mutant lacking the CTP regions of the eCG β-subunit is not clear. We suggest that each glycosylated site of eCG plays a specific role in secretion.

The molecular weight of purified eCG produced from the equine placenta during early pregnancy was 44–50 kDa protein. In this study, the eCG band was broadly detected at approximately 40–45 kDa inn the CHO-S cells. These results are consistent with those of our previous studies, which suggested that the size of eCG was approximately 40–45 kDa in attached CHO-K1 cells [13]. Additionally, doublet protein with 46 and 44 kDa was detected in the COS-7 cells [4]. The molecular weight of rec-eCGβ/α expressed in the Sf9 insect cells was approximately 45 kDa. The herodimeric eCG exhibited an upper band at 45 kDa and a lower band at approximately 38–40 kDa [5]. However, the molecular weight of the main band of eCG in the transgenic rabbit was approximately 35 kDa [45]. Thus, the molecular weight of rec-eCGβ/α in mammalian cultured cells (CHO-K1 and COS7) was similar to that in the CHO-S cells. In this study, the analysis of glycosylation site mutants confirmed that the loss of glycosylation sites markedly decreases the molecular weight of the N-linked and O-linked glycosylation site deletion mutants.

Several studies have reported that the in vitro biological activity of CG mutants lacking glycosylation sites is 5**–**10-fold lower than that of CG with glycosylation sites [30, 46–48]. The glycosylation site at Asn^52^ of the hCG α-subunit was critical for LH-like activity and signal transduction of hCG as evidenced by decreased steroidogenic and cAMP responses of Asn^52^ mutants [29]. However, the signal transduction activity of mutants lacking CTP region of the hCG β-subunit was unaffected [40]. The O-linked glycosylation sites (115–145 residues) at hCG β-subunit are not involved in receptor binding and signal transduction in MA-10 Leydig tumor cells [49, 50]. However, residues 101–145 were critical for signal transduction during progesterone production as the truncation of this region markedly downregulated the activity in the MA-10 Leydig tumor cells [51]. Previously, we had reported that heterodimeric rec-eCGα/β and single-chain rec-eCGβ/α exhibited LH-like activity similar to that of native eCG using an in vitro bioassay with primary rat Leydig cells, and granulosa cells, respectively [17, 34], as well as using PathHunter parental cells expressing eLH/CGR [52]. We suggest that Asn^56^ of the eCG α-subunit is critical for signal transduction and that the CTP region (114–149 residues) of the eCG β-subunit is not essential for signal transduction in CHO-K1 cells expressing rLH/CGR.

Various studies have used site-directed mutagenesis to identify the glycosylation sites on FSH [48, 53, 54]. One study examining the biological activity of rec-hFSH mutants revealed that the FSH activity of a mutant lacking the glycosylation site at Asn^52^ of α-subunit decreased by 72% when compared with that of rec-hFSH-wt in the Y-1 cell line expressing hFSHR, which indicate that the glycosylation site at Asn^52^ was critical for signal transduction [31]. In the Sertoli cells of immature rats (10-day old male), the removal of the glycosylation site at Asn^52^ decreased the activity to 26% of that of wild-type [30]. However, the other glycosylation sites at Asn^78^ of the α-subunit and Asn^7^ and Asn^24^ of the β-subunit were not involved in signal transduction. In the rat granulosa cell system, the signal transduction activity of FSH lacking the glycosylation site at Asn^52^ of the α-subunit was markedly downregulated, while that of FSH lacking the glycosylation sites at Asn^78^ of α-subunit and Asn^7^ and Asn^24^ of β-subunit were slightly downregulated [32]. The analysis of eFSHαΔ56/β revealed that eFSH glycosylation sites have a major role in signal transduction in primary rat granulosa cells [33]. These findings suggest that glycosylation sites play a pivotal role in determining the biological activity of eCG. The EC_50_ and Rmax values of rec-eCGβ/αΔ56 were approximately 24.3% and 33.3% of those of rec-eCGβ/α-wt. The glycosylation sites at Asn^56^ of the eCG α-subunit (Asn^52^ of the hCG α-subunit) are necessary for signal transduction. The FSH-like cAMP response of the hFSHβ-hCGβ linker-hFSHα mutant in which the CTP region (118–145 aa) of the hCG β-subunit was inserted between the hFSH β-subunit and the α-subunit is similar to that of wild-type in the HEK-293 cells expressing FSHR [40, 54, 55]. However, the mutant exhibited enhanced in vivo potency and half-life in the circulation.

Previously, we had reported that rec-eCGβ/α exhibits dose-dependent FSH-like activity in cells expressing rFSHR [13, 17]. The findings of this study are consistent with those of previous studies, which reported that the mutation at Asn^56^ of the α-subunit resulted in decreased cAMP response in cells expressing rFSHR. Thus, the glycosylation site at Asn^56^ of α-subunit plays a pivotal role in FSH-like activity. Additionally, the contribution of α-subunit to FSH-like activity is higher than that of the CTP region of the β-subunit. However, eCGβ/α-wt, eCG β/αΔ56, eCGβ-D/α, and eCG β-D/αΔ56 mutants exhibited similar effects on the ovulation rates of oocytes in vivo with non-functional oocytes accounting for only 2% [17]. Therefore, we suggest that the FSH-like activity of eCG varies in vivo and in vitro even though the mechanisms are not fully understood, except for the role of oligosaccharides. Previous studies have revealed that natural eCG and eLH α-subunit contain different oligosaccharide structure at Asn^56^. eLH is primarily expressed as seven variants of a monoantennary structure, while eCG is expressed in two forms (a sialylated biantennary oligosaccharide or a lactosamine variant) [56]. Three eCG forms (eCG-L, eCG-M, and eCG-H) with different molecular weight are generated based on the O-glycosylation patterns [57]. eCG-H exhibits significantly lower receptor-binding activities than eCG-L and eCG-M. Previously, we had demonstrated that O-linked oligosaccharides of rec-eCG produced from CHO-K1 cell does not modified as evidenced by the lack of change in molecular weight of rec-eCG upon treatment with enzymes that digest O-linked glycan [58]. Our data cannot exclude that the mutant of the glycosylation site at the Asn^56^ of the α-subunit and the deleted mutant of the CTP-region including O-glycosylation sites is induced the structural alteration for rec-eCG proteins. Thus, we suggest that the O-glycosylation motif should individually mutate to explain the systematic analysis result. These findings indicate that the dual activities of the eCG molecule could be regulated by modulating the glycosylation sites and structures.

## Conclusions

The findings of this study suggest that rec-eCGβ/α exhibits potent biological activity in CHO-K1 cells expressing rLH/CGR and rFSHR. rec-eCGβ/α-wt was efficiently secreted into the culture medium of CHO-S cells on day 1 post-transfection. CTP (aa 114–149) of the eCG β-subunit containing approximately 12 O-glycosylation sites was critical for the early secretion of eCG in the CHO-S cells transfected with a mammalian expression vector system. The glycosylation site at Asn^56^ of the α-subunit plays an important role in both LH-like and FSH-like activities of eCG. In particular, the LH-like activity of the mutant lacking of the eCG β-subunit was unaffected but the activity was completely inhibited in the double mutant. The FSH-like activity of rec-eCGβ/αΔ56 markedly decreased, whereas that of rec-eCGβ-D/α was not affected. The cAMP response of rec-eCGβ-D/α was similar to that of eCGβ/α-wt. The oligosaccharides at Asn^56^ of the α-subunit and O-linked oligosaccharides of the β-subunit exhibit differential roles in signal transduction and secretion of rec-eCG. A rec-eCGβ/α mutant exhibiting only FSH-like activity for ovulation without LH-like has been constructed using DG44 cells. Thus, further studies are needed to examine the functional significance of rec-eCGs in equids. Future studies must elucidate the functional mechanisms that regulate the roles of eCG in the ovary and testis of equine species.

## Methods

### Materials

The cloning vector (pGEMTeasy) was purchased from Promega (Madison, WI, USA). The pcDNA3 mammalian expressing expression vector, CHO-S cells, FreeStyle MAX reagent, FreeStyle CHO expression medium, anti-*myc* antibody, Lipofectamine 2000, and antibiotics were purchased from Invitrogen Corporation (Carlsbad, CA, USA). The oligonucleotides used in this study were synthesized by Genotech (Daejeon, Korea). Restriction enzymes, PCR reagents, and DNA ligation kit were purchased from Takara (Shiga, Japan). CHO-K1 cells were obtained from the Japanese Cancer Research Resources Bank (Tokyo, Japan). Ham’s F-12 medium, Opti-MEM I, and serum-free CHO-S-SFM II were purchased from Gibco BRL (Grand Island, NY, USA). Fetal bovine serum (FBS) was obtained from Hyclone Laboratories (Logan, UT, USA). Centriplus centrifugal filter devices were purchased from Amicon Bio separations (Billerica, MA, USA). The deglycosylation kit (PNGase F) was purchased from New England Biolabs (Ipswich, MA, USA). The Lumi-Light western blot kit was purchased from Roche (Basel, Switzerland). The QIAprep-Spin plasmid kit was purchased from Qiagen. Inc. (Hilden, Germany). Disposable spinner flasks were obtained from Corning Inc. (Corning, NY, USA). The PMSG enzyme-linked immunosorbent assay (ELISA) kit was purchased from DRG International Inc. (Mountainside, NJ, USA). The cAMP Dynamic 2 immunoassay kit was purchased from Cisbio (Dodolet, France). All other reagents used were purchased from Sigma-Aldrich Corp. (St. Louis, MO, USA).

### Construction of single-chain eCGβ/α mutants

To construct single-chain eCG, the cDNA encoding the full-length eCG β-subunit was fused with that encoding α-subunit using overlapping PCR mutagenesis as previously reported [17]**.** Site-directed mutagenesis was performed to mutate the glycosylation sites to examine the functional importance of oligosaccharides in both LH-like and FSH-like activities of eCG. The same method was used to add a *myc*-tag (10 amino acids; Glu-Gln-Lys-Leu-Ile-Ser-Glu-Glu-Asp-Leu) between the first and second amino acids of the mature protein in the eCG β-subunit as previously reported [19]**.** The PCR fragments encoding single-chain eCG were digested with *Eco*RI and *Sal*I and cloned into the *Eco*RI and *Xho*I sites of the eukaryotic expression vector pcDNA3 (designated as pcDNA3-eCGβ/α). Additionally, cDNAs encoding the single-chain eCGβ/α cloned into the pcDNA3 vector were used as templates to construct the mutants in which Asn (AAC) at the glycosylation site was substituted with Gln (CAG) or to delete the CTP (115–149 aa) comprising approximately 12 O-glycosylation sites of the eCG β-subunit as previously reported [13]. Schematic diagrams of single-chain eCG/mutants are shown in Fig. 1. The orientation of the constructs was confirmed through restriction mapping. Finally, this vector was subjected to sequence to confirm the Kozak site, *myc*-tag, and PCR errors. In total, four expression vectors were constructed (designated as pcDNA3-eCGβ/α, pcDNA3-eCGβ/αΔ56, pcDNA3-eCGβ-D/α, and pcDNA3-eCGβ-D/αΔ56) as previously reported [13]**.**

### Transient transfection and production of rec-eCGβ/α mutants in CHO suspension cell

The mammalian vectors encoding eCGβ/α mutants were transfected into CHO-S cells using the FreeSytle MAX reagent, following the manufacturer’s instructions. Briefly, the CHO-S cells were cultured in FreeStyle CHO expression medium at a density of 1 × 10^7^ cells/30 mL for 3 days. One day before transfection, the cells were passaged at a density of 5–6 × 10^5^ cells/mL with CHO expression medium (125 mL) in a disposable spinner flask. On the day of transfection, the cell density was approximately 1.2–1.5 × 10^6^ cells/mL. Next, DNA (160 μg) was mixed gently in 1.2 mL of OptiPRO serum-free medium. FreeSytle MAX reagent (160 μL) was mixed gently in 1.2 mL of the OptiPRO serum-free medium. The two media were mixed and incubated for 5 min at room temperature. The complex (2.4 mL) was added to each cell suspension flask. For the recombinant protein assay, 2 mL of the culture medium was collected on days 0, 1, 3, 5, 7, and 9 post-transfection. The culture medium was centrifuged at 15,000 rpm at 4 °C for 10 min to remove cell debris. The supernatant was stored at − 80 °C until analysis. The culture supernatant collected from day 9 was subjected to freeze drying or concentration. To measure the biological activity of the concentrated samples, the samples were mixed 10–20 times and subjected to ELISA or western blotting.

### Hormone quantitation of rec-eCGβ/α proteins

The rec-eCG proteins were quantified using PMSG ELISA with anti-PMSG monoclonal antibody and horseradish peroxidase (HRP)-conjugated antibody and TMB substrate, following the manufacturer’s instructions. The culture medium (100 μL) was dispensed into the wells of 96-well plates coated with a monoclonal antibody against a unique antigenic site on the eCG molecule. The samples were incubated for 60 min at room temperature, followed by incubation with 100 μL of HRP-conjugated secondary antibody. Next, the samples were washed five times with 300 µL of distilled water and incubated with the substrate solution (100 μL) for 30 min at room temperature. Further, 50 μL of stop solution was added. The absorbance of the mixture at 450 nm was measured within 30 min using a plate reader. Finally, I IU was assumed to be 100 ng according to the conversion factor of the suggested assay protocol.

### Western blotting and enzymatic digestion of N-linked oligosaccharides

Equal amounts of concentrated sample (10 µg) were subjected to reducing sodium dodecyl sulfate–polyacrylamide gel electrophoresis (SDS-PAGE) with a 12.5% gel**.** The proteins were transferred onto a polyvinylidene difluoride membrane (0.2 µm) using a Bio-Rad Mini Trans-Blot electrophoresis cell. The membrane was washed with 1X Tris-buffered saline containing Tween 20 (TBS-T) and incubated with the anti-myc antibody (1:5000). Next, the membrane was incubated with the HRP-conjugated goat anti-mouse IgG (1:3000). The membrane was then incubated with 2 mL of the Lumi-Light substrate solution for 1 min. The substrate solution was removed and the membrane was placed on the Saran wrap, covered with a second piece of the Saran wrap, and exposed on X-ray films for 1–10 min. Additionally, glycans in the rec-eCG proteins were removed using peptide-N-glycanase F. The molecular weights of the proteins were analyzed using western blotting. To remove all N-linked glycans, rec-eCG proteins (20 μg) were incubated with peptide-N-glycanase F [1 µL enzyme (2.5 u/mL) + 10 µL reaction buffer (2 µL 10X Glyco buffer + 2 µL 10% NP-40, and 6 µL distilled water)] for 24 h at 37 °C. The reaction was stopped by boiling for 10 min and the samples were subjected to SDS-PAGE and western blotting.

### Construction of rLH/CGR and rFSHR expression vector

rLH/CGR and rFSHR were cloned into the pcDNA3 expression vector and transfected into CHO-K1 cells as previously reported [19]. The PCR fragments were cloned into the pcDNA3 mammalian expression vector at the *Eco*R1 and *Xho*1 sites (designated as pcDNA3-rLH/CGR and pcDNA3-rFSHR). The receptor cDNAs were also subcloned into the eukaryotic expression vector pCORON 1000SP VSV-G tag for transfection (designated as pVSVG-rLH/CGR and pVSVG-rFSHR) [58].

### Transient transfection into the CHO-K1 cells

Transfection of CHO cells was performed using the liposome transfection method as previously described [43]. The CHO cells were cultured in growth medium [Ham’s F-12 media supplemented with penicillin (50 U/mL), streptomycin (50 µg/mL), glutamine (2 mM), and 10% FBS] until 80%–90% confluency in six-well plates and transfected with plasmid DNAs using Lipofectamine reagent. The diluted DNA was mixed with Lipofectamine reagent and the cells were incubated with the DNA-Lipofectamine complex for 5 h. Next, the cells were incubated with CHO growth medium supplemented with 20% FBS. The medium was replaced at 24 h post-transfection. The transfected cells were seeded in 384-well plates (10^4^ cells per well) and cAMP production was analyzed at 48–72 h post-transfection.

### cAMP analysis using homogenous time-resolved foster resonance energy transfer (HTRF) assays

The cAMP levels in the CHO-K1 cells expressing rLH/CGR and rFSHR were measured using cAMP Dynamics 2 competitive immunoassay kits (Cisbio Bioassays, Codolet, France) as described previously [43]. The assay was performed using a cryptate-conjugated anti-cAMP monoclonal antibody and d2-labeled cAMP. The cells transfected with rLH/CGR and rFSHR were seeded in 384-well plates (10,000 cells per well). The standard samples were prepared to cover an average concentration cAMP range of 0.17–712 nM (final concentration of cAMP per well). MIX was added to the cell dilution buffer to prevent cAMP degradation. To each well, 5 µL of compound medium buffer containing rec-eCG mutants was added. The plate was sealed and incubated for cell stimulation at room temperature for 30 min. The samples were then incubated with cAMP-d2 (5 µL) and anti-cAMP-cryptate (5 µL) for 1 h at room temperature. cAMP was detected by measuring the decrease in HTRF energy transfer (665 nm/620 nm) using an Artemis K-101 HTRF microplate reader (Kyoritsu Radio, Minato-Ku, Japan). The specific signal Delta F (energy transfer) is inversely proportional to the concentration of cAMP in the standard or sample. The results were calculated from the 665 nm/620 nm ratio and expressed as Delta F % (cAMP inhibition).

$${\text{Delta F}}\% = \left( {{\text{Standard or sample ratio}} - {\text{sample negative}}} \right) \times {1}00/{\text{ratio negative}}.$$

The Delta F% values were calculated using GraphPad Prism software (GraphPad, Inc., La Jolla, CA, USA).

### Data and statistical analysis

The sequences were compared using the Multalin interface multiple sequence alignment tool. The data of the dose–response curve were generated from experiments performed in duplicates. The cAMP levels in all transfected cells were subtracted from those in the mock-transfected cells. GraphPad Prism 6.0 was used to examine the cAMP production and EC_50_ values and analyze the stimulation curve. The curves fitted in a single experiment were normalized to the background signal measured for the mock-transfected cells (Figs. 4, 5). The results are expressed as the mean ± standard error of mean from three independent experiments. The means of multiple groups were compared using one-way analysis of variance, followed by Tukey’s post-hoc tests with GraphPad Prism 6.0. The differences were considered significant at *p* < 0.05.

## Data Availability

The datasets used and analyzed in the current study are available from the corresponding author on reasonable request.

## Abbreviations

eCG: Equine chorionic gonadotropin
LH/CGR: Luteinizing hormone/chorionic gonadotropin receptor
FSHR: Follicle-stimulating hormone receptor
LH: Luteinizing hormone
FSH: Follicle-stimulating hormone
CTP: Carboxyl-terminal peptide
CHO-S cells: Chinese hamster ovary suspension cells
hCG: Human chorionic gonadotropin
rec-eCG: Recombinant-eCG
rLH/CGR: Rat luteinizing hormone/chorionic gonadotropin receptor
rFSHR: Rat follicle-stimulating hormone receptor
